# Impact of Prenatal and Postnatal Exposure to Endocrine Disrupter DDT on Adrenal Medulla Function

**DOI:** 10.3390/ijms23094912

**Published:** 2022-04-28

**Authors:** Nataliya V. Yaglova, Sergey S. Obernikhin, Dibakhan A. Tsomartova, Valentin V. Yaglov, Svetlana V. Nazimova, Elina S. Tsomartova, Ekaterina P. Timokhina, Elizaveta V. Chereshneva, Marina Y. Ivanova, Tatiana A. Lomanovskaya

**Affiliations:** 1 Laboratory of Endocrine System Development, Research Institute of Human Morphology, FSBSI, Petrovsky National Research Centre of Surgery, 119991 Moscow, Russia; ober@mail.ru (S.S.O.); dtsomartova@mail.ru (D.A.T.); vyaglov@mail.ru (V.V.Y.); pimka60@list.ru (S.V.N.); tselso@yandex.ru (E.S.T.); rodich@mail.ru (E.P.T.); 2 Department of Histology, Cytology, and Embryology, Federal State Funded Educational Institution of Higher Education I.M. Sechenov First Moscow State Medical University, 119435 Moscow, Russia; yelizaveta.new@mail.ru (E.V.C.); ivanova_m_y@mail.ru (M.Y.I.); tatyana_80_80@inboxl.ru (T.A.L.)

**Keywords:** epinephrine, adrenal medulla, endocrine disruption, DDT, secretion, mitochondria, tyrosine hydroxylase, chromaffin cells, fine structure

## Abstract

Epinephrine is the most abundant catecholamine hormone, produced by the nervous system and adrenal glands. Endocrine disruption of epinephrine synthesis, secretion and signaling is less studied than steroid and thyroid hormones. Dichlorodiphenyltrichloroethane (DDT) is recognized as one of the most prominent environmental contaminants with a long half-life. It is a potent endocrine disrupter affecting sex steroid, mineralocorticoid, glucocorticoid and thyroid hormone production. Exposure to low doses of DDT is universal and begins in utero. Therefore, we studied adrenal medulla growth and function in male Wistar rats exposed to low doses of DDT during prenatal and postnatal development until puberty and adulthood, as well as rats exposed to DDT since the first day of postnatal development. All the exposed rats demonstrated lowered epinephrine blood levels, gradually reducing with age. DDT was found to inhibit the synthesis of tyrosine hydroxylase and affect the mitochondrial apparatus of epinephrine-producing cells during puberty and even after maturation. Low-dose exposure to DDT from birth resulted in more pronounced changes in adrenomedullary cells and a more profound decrease (up to 50%) in epinephrine secretion in adult rats. Prenatal onset of exposure demonstrated a mild effect on epinephrine-producing function (30% reduction), but was associated with lower rate of adrenal medulla growth during maturation and 25% smaller adrenal medullar size in adult rats. All subjects exposed to low doses of DDT failed to develop adaptive changes and restore proper epinephrine production. These results indicate a dysmorphogenetic effect of prenatal exposure and disruption of secretory function of adrenal chromaffin cells by postnatal exposure to DDT.

## 1. Introduction

Endocrine disrupting chemicals are known to interfere with hormone production, hormone-receptor interactions and hormone signaling [1,2]. The list of endocrine disrupters is constantly expanding, as is the list of the properties of identified disrupters. Low-dose exposure to environmental pollutants which disrupt endocrine function is a growing threat due to their worldwide dissemination and long half-lives. The insecticide dichlorodiphenyltrichloroethane (DDT) is recognized as one of the most prominent environmental contaminants [3]. Many countries banned DDT in 1970s, but in 2006, its use was reintroduced by the World Health Organization for malaria and other vector-borne disease control in some countries [4]. Biomonitoring studies over the last decade have shown that DDT still remains the most-widespread endocrine disrupting chemical [5,6,7,8]. The presence of DDT metabolites in 99.9% of maternal and child blood samples taken in European countries indicates that rates of low-dose exposure are not diminishing [9]. Because DDT is a small lipophilic molecule, it easily crosses blood–tissue barriers, including placental barriers [10]. Numerous investigations have revealed effects on all steroid (male and female sex steroids, mineralocorticoids, and glucocorticoids) [11,12,13], amino acids-derived (thyroid) [14,15,16] and some protein hormones by DDT [17]. 

The ability of DDT to disrupt catecholamines secretion is less well studied. Epinephrine is the most abundant catecholamine hormone produced by the nervous system and adrenal glands. It plays a pivotal role in acute stress response, regulates the metabolism of carbohydrates and lipids and acts as a neurotransmitter, providing proper functioning of somatic organs [18]. Adrenal medulla provides up to 80% of the epinephrine blood pool, which is why normal function of adrenal chromaffin cells is crucial for epinephrine supply. Epinephrine is a derivative of amino acid tyrosine, like thyroid hormones. In our previous studies, we observed impaired catecholamine production after developmental exposure to DDT [19]. These findings compelled us to elucidate the mechanisms of disruption of adrenal medulla hormone secretion. It is well known that exposure to DDT begins in utero and lasts throughout life, as the main sources of DDT are daily consumed food products [20]. The outcomes of early life DDT exposure for adrenal medulla function are not known. The role of prenatal or postnatal exposure, time period when signs of disruption begin, duration of manifestations, and compensatory and adaptive changes require elucidation. We focused on adrenal medulla development and function in rats exposed to low-doses of DDT during prenatal and postnatal development until puberty and adulthood, as well as rats exposed to DDT since the first day of postnatal development. 

## 2. Results

### 2.1. Changes in Epinephrine Plasma Levels

**Figure 1:**
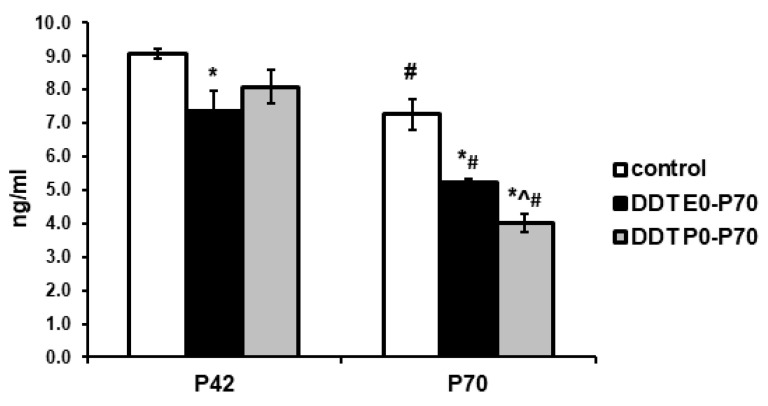
Epinephrine plasma concentration in the control and o,p-DDT exposed rats. Data are shown as mean ± S.E.M. P, day of postnatal development; *p* < 0.05 compared to the control (*), compared to the group DDT E0-P70 (^), compared to P42 (#).

Epinephrine plasma levels in prenatally and postnatally exposed rats during puberty were significantly lower than in the control animals. Postnatally exposed rats demonstrated smaller decrease of epinephrine levels in pubertal age (Figure 1).

Epinephrine plasma contents in the control rats showed a 20% reduction after maturation. All DDT-exposed rats demonstrated similar but more profound changes in epinephrine production. In prenatally and postnatally exposed rats, age-related reduction of epinephrine plasma levels was about 30%. Postnatally exposed rats exhibited a two-fold decrease in epinephrine production. All DDT-exposed rats had significantly lower epinephrine plasma levels after puberty, but maximal reduction of epinephrine production was observed in postnatally exposed rats (Figure 1). 

### 2.2. Changes in Histology of Adrenal Medulla

**Figure 2:**
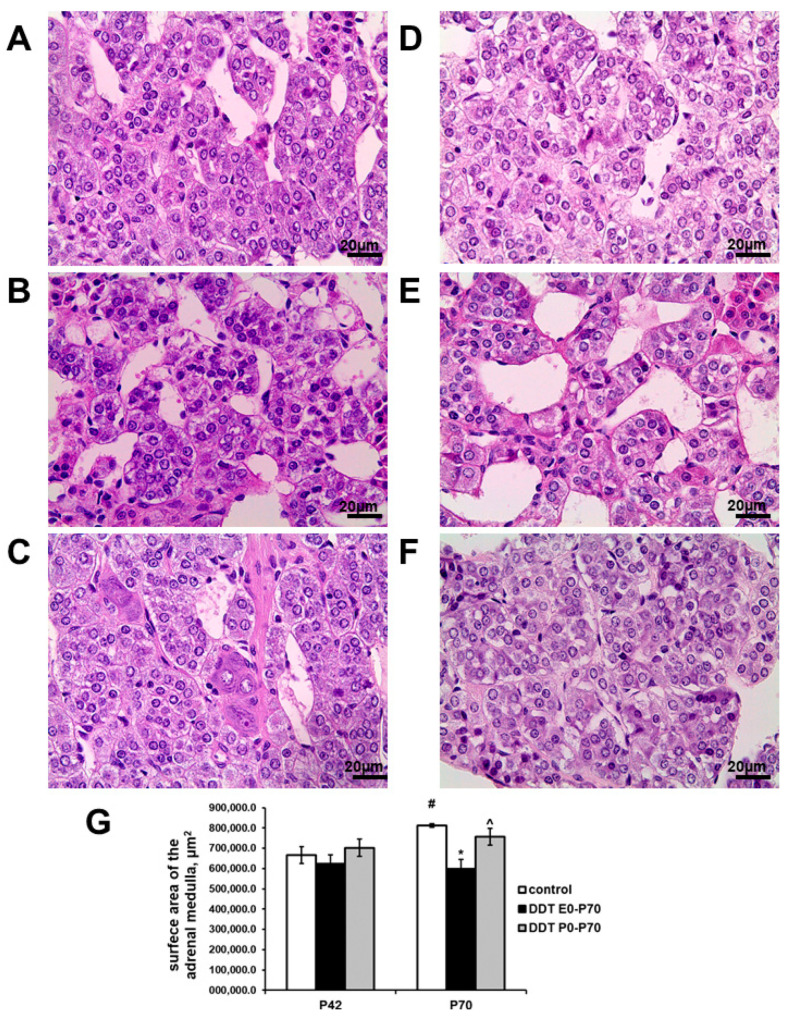
Changes in the histology of the adrenal medulla after developmental exposure to low doses of o,p’-DDT. Histology of the adrenal medulla of the control rats (**A**), prenatally and postnatally exposed to DDT rats (**B**) and postnatally exposed to DDT rats (**C**) in pubertal period (P42), of the control rats (**D**), prenatally and postnatally exposed to DDT rats (**E**) and postnatally exposed to DDT rats (**F**) after puberty (P70). Magnification 400. (**G**) Surface area of the adrenal medulla. Data are shown as mean ± S.E.M. P, day of postnatal development; *p* < 0.05 compared to the control (*), compared to the group DDT E0-P70 (^), compared to P42 (#).

In prenatally and postnatally exposed rats, adrenal medulla exhibited typical histology. Chromaffin cells had enlightened nuclei and basophilic staining of cytoplasm, like in the control adrenals (Figure 2A,B). Microcirculatory vessels were well developed. No microcirculatory disorders were observed (Figure 2B). The surface area of the medulla in the equatorial sections was smaller than the control values at pubertal age, but these differences did not reach statistical significance (Figure 2G). Postnatally exposed rats also demonstrated normal development of adrenal medulla and no visual changes in its structure (Figure 2C,G).

After puberty, the adrenal medulla of the control rats had significantly increased surface area compared to samples taken from prepubescent rats (Figure 2G). The chromaffin cells had less intensive basophilic staining than in pubertal age (Figure 2D). Unlike the control, prenatally and postnatally exposed rats showed no age-related increase in adrenal medulla size. Their adrenal medulla surface area was 25% smaller than the control values (Figure 2G). Postnatally exposed rats showed a slight increase in medulla size with age, although the surface area of their adrenal medulla did not significantly differ from the control (Figure 2G). No principal changes in adrenal medulla histology of the DDT-exposed rats were found (Figure 2E,F).

### 2.3. Changes in Fine Structure of Adrenal Chromaffin Cells

**Figure 3:**
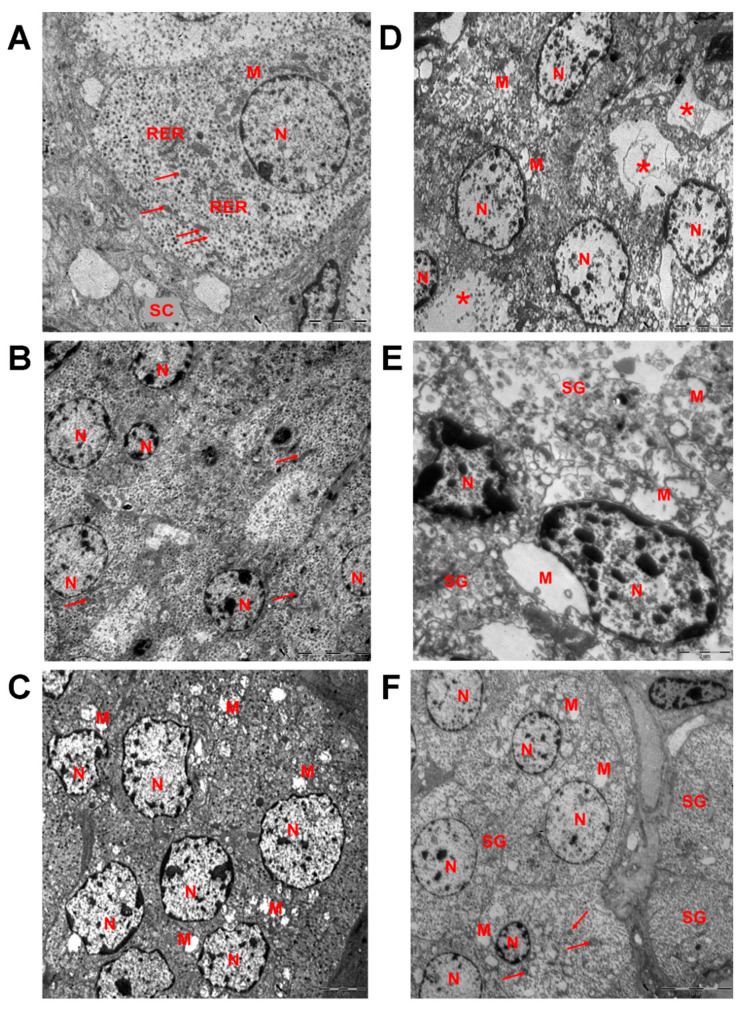
Epinephrine-producing cells of the adrenal medulla of the control and the exposed to low doses of o,p’-DDT rats. Transmission electron microscopy. Fine structure of the epinephrine-producing cells of the control rats (**A**), prenatally and postnatally exposed to DDT rats (**B**) and postnatally exposed to DDT rats (**C**) in pubertal period (P42), of the control rats (**D**), prenatally and postnatally exposed to DDT rats (**E**) and postnatally exposed to DDT rats (**F**) after puberty (P70). M, mitochondria with swollen matrix; N, nucleus; SG, secretory granules, RER, rough endoplasmatic reticulum; arrows point unaffected mitochondria, asterisks–foci of devastation of cytoplasm. Scale bars 5 µm for (**A**,**C**,**D**), 10 µm for (**B**,**E**), 2 µm for (**E**).

In the control rats of pubertal age, the adrenal medulla was composed of chromaffin epinephrine- and norepinephrine-producing cells associated with blood vessels, nerve and connective tissue elements. Among the chromaffin cells, epinephrine-producing cells prevailed, while norepinephrine-producing cells were less numerous. Epinephrine-producing cells were polyhedral in shape, containing single round nuclei with well-defined nucleolus. The cytoplasm had low electron density. It contained rough endoplasmatic reticulum, Golgi complex and numerous mitochondria. Mitochondria were elongated in shape and exhibited transverse cristae and matrix of moderate electron density. Most of the mitochondria were located around the cell nucleus. Most of the mitochondria were located around the cell nucleus. The rest were found in the vicinity of the outer membrane or were associated with rough endoplasmic reticulum. Secretory granules were abundant. They were round in shape, membrane-bound and had electron-dense content (Figure 3A).

**Figure 4:**
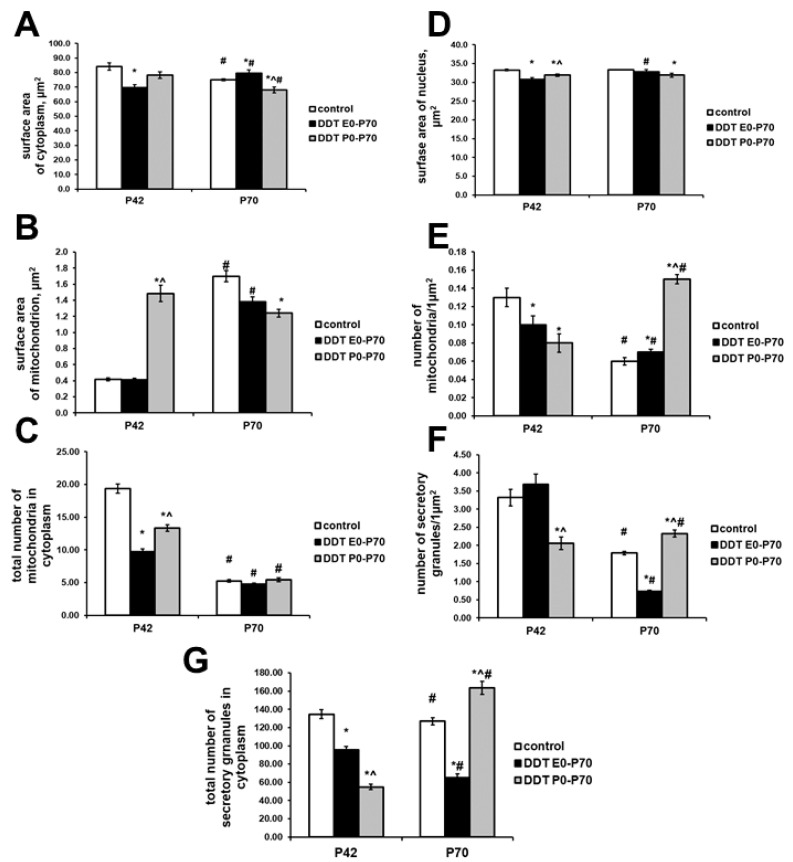
Changes in fine structure of the epinephrine-producing cells after developmental exposure to low doses of o,p’-DDT. Surface area of cytoplasm (**A**), surface area of nucleus (**B**), surface area of mitochondrion (**C**), number of mitochondria per 1 μm^2^ of cytoplasm (**D**), total number of mitochondria in cell (**E**), number of secretory granules per 1 μm^2^ of cytoplasm (**F**) total number of mitochondria in cell (**G**). Data are shown as mean ± S.E.M. P, day of postnatal development; *p* < 0.05 compared to the control (*), compared to the group DDT E0-P70 (^), compared to P42 (#).

Prenatally and postnatally exposed rats showed typical adrenal medulla ultrastructure. However, epinephrine-producing cells were smaller in size, and both nucleus and cytoplasm area were diminished (Figure 4A,B). No changes in the fine structure of mitochondria were observed (Figure 3B and Figure 4C). The number of mitochondria per 1 µm^2^ of cytoplasm and total number of mitochondria in cell were significantly reduced (Figure 4D,E). Cytoplasm contained numerous secretory granules with highly electron-dense material (Figure 3B). Quantification of secretory granules revealed no differences in their number per 1 µm^2^ of cytoplasm, but found 30% reduction of their total content in cells (Figure 4F,G).

In postnatally exposed rats, epinephrine-producing cells demonstrated smaller reduction of cytoplasm and nucleus surface area than the previous group (Figure 4A,B). The fine structure of chromaffin cells was distinct from the control and prenatally and postnatally exposed rats. Mitochondria were significantly enlarged, had low dense swollen matrix and disrupted cristae (Figure 3C and Figure 4C). Their number per 1 µm^2^ of cytoplasm was reduced compared to the control (Figure 4D). Total number of mitochondria in cells was also smaller than in the control, but higher than in the prenatally and postnatally exposed rats (Figure 4D,E). Secretory granules were scattered in cytoplasm. They had moderate electron-dense content (Figure 3C). Secretory granules content per 1 µm^2^ of cytoplasm and per cell were decreased and had the lowest values among the groups (Figure 4F,G).

After puberty, the control rats showed some changes in the fine structure of epinephrine-producing cells. A smaller reduction of cytoplasm surface area was found (Figure 4A). No changes were revealed in structure of nuclei (Figure 3D and Figure 4B). Significant changes in mitochondria apparatus were observed. Mitochondrial size was 4 times larger than at puberty (Figure 4C). Most mitochondria had swollen matrix (Figure 3D). However, number of mitochondria per 1 µm^2^ of cytoplasm decrease only 2 times (Figure 4D). Total number of mitochondria in cytoplasm was 3.5 times reduced compared to pubertal age (Figure 4E). Mitochondria, rough endoplasmatic reticulum and Golgi complex demonstrated paranuclear localization. Secretory granules were located unevenly in the cytoplasm. There were areas of compact arrangement of granules in cytoplasm and areas that did not contain granules and organelles (Figure 3D). Their number per 1 µm^2^ and total number in cytoplasm were smaller than in pubertal age (Figure 4F,G).

Epinephrine-producing cells of prenatally- and postnatally-exposed rats enlarged with age. Their surface area of cytoplasm was larger than in the control group after puberty (Figure 4A). The size of the nuclei, although it increased with age, did not exceed the values of the control group (Figure 4B). Mitochondrial apparatus demonstrated similar to the control age-dependent changes–enlargement of mitochondria due to swelling of matrix, decrease of their content in cell, and paranuclear localization (Figure 3E and Figure 4C,E). Focal devastation of cytoplasm was often observed. Number of secretory granules per 1 µm^2^ of cytoplasm and their total number per cell reduced with age and were significantly diminished compared to the control (Figure 4F,G). Some secretory granules had dissolved internal cores (Figure 3E).

Postnatally exposed rats also exhibited significant changes in fine structure of epinephrine-producing adrenomedullary cells. The size of cytoplasm reduced with age and was the smallest among postpubertal rats of compared groups (Figure 4A). The size of nuclei was also smaller than in the control (Figure 4B). Examination of mitochondrial apparatus revealed both irregular in shape large mitochondria with swollen matrix and disrupted cristae and smaller in size mitochondria with normal structure of matrix and cristae (Figure 3F). The average size was mitochondria decreased with age and was the smaller than in the control and prenatally and postnatally exposed rats of the same age (Figure 4C). Despite the fact that the number of mitochondria per 1 µm^2^ of cytoplasm increased and exceeded the control values, their total number decreased and did not differ from the control (Figure 4D,E). Mitochondria were usually observed in paranuclear area (Figure 3F). Rough endoplasmatic reticulum and Golgi complex were less prominent. Significant changes were observed in content of secretory granules. Their number per 1 µm^2^ of cytoplasm and total number per cell increased with age unlike the control rats and were higher than in the control group and group of prenatal and postnatal exposure (Figure 4F,G). Secretory granules were diffusely distributed in the cytoplasm (Figure 3F). Dissolved internal core and enlightened area under the membrane of granule were frequently found.

### 2.4. Changes in Tyrosine Hydroxylase Production

**Figure 5:**
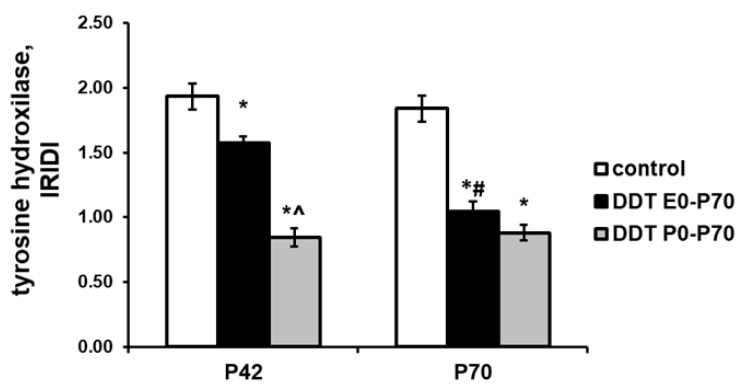
Changes in immunoreactivity intensity distribution index (IRIDI) for tyrosine hydroxylase in epinephrine-producing cells after developmental exposure to low doses of o,p’-DDT. Data are shown as mean ± S.E.M. P, day of postnatal development; *p* < 0.05 compared to the control (*), compared to the group DDT E0-P70 (^), compared to P42 (#).

An immunohistochemical evaluation revealed extremely high tyrosine hydroxylase content with diffuse distribution in the cytoplasm of adrenomedullary chromaffin cells in the control rats of pubertal age. An evaluation of tyrosine hydroxylase in prenatally and postnatally exposed rats revealed a diminished enzyme content. Postnatally exposed rats exhibited the most pronounced decrease in tyrosine hydroxylase production (Figure 5).

After puberty, the chromaffin cells of the control rats demonstrated a slight, insignificant decrease of tyrosine hydroxylase content (Figure 5). In prenatally and postnatally exposed rats, the age-related decrease was more profound, and the tyrosine hydroxylase content was half than in the control (Figure 5). Postnatally exposed rats showed no changes in tyrosine hydroxylase production with age, and the tyrosine hydroxylase content in the cytoplasm of chromaffin cells was minimal among the compared groups (Figure 5).

## 3. Discussion

The present findings demonstrate impaired epinephrine production in DDT-exposed rats and some differences in adrenal medulla function depending on the period of ontogeny when of exposure began. We assessed the morphology and function of the adrenal medulla in a period of active growth (puberty) and after termination of adrenal growth, which usually happens after puberty on the 70th day of postnatal development [21]. This required assessments and differentiation of age-dependent and DDT-provoked alterations. 

It is well known that adrenomedullary chromaffin cells are a major source of circulating epinephrine, and disruption of adrenal medulla function negatively influences epinephrine blood levels [22]. Epinephrine plasma levels were found to decrease after puberty in the control rats. An evaluation of the DDT-exposed rats revealed both age-related and DDT-induced alterations in epinephrine production. Both postnatally and prenatally and postnatally exposed rats exhibited similar changes—age-related decrease and attenuated epinephrine production in both pubertal and postpubertal periods. This indicated that endocrine disruption of adrenomedullary function did not affect age-dependent patterns of epinephrine release. As such, our investigations of the mechanisms underlying disruption by low-dose exposure to DDT epinephrine production required elucidation of the mechanisms behind age-related down-regulation of epinephrine production in the control rats. 

It is noteworthy that the control rats showed decreased epinephrine production after maturation despite an increase in adrenal medulla size. This indicated that rate of epinephrine synthesis did not depend on number of chromaffin cells in the medulla. Tyrosine hydroxylase is a key enzyme which regulates the rate of epinephrine production, as well as a well-recognized marker of differentiated chromaffin cells [23]. We found abundant tyrosine hydroxylase in the cytoplasm of adrenomedullary cells of the control rats and no significant changes in its expression with age. This suggests that other factors may be involved in determining the functional activity of cells, in addition to the rate of synthesis. The most likely mechanism for reduced epinephrine production was an altered secretory apparatus of the adrenomedullary cells. Chromaffin cells have been shown to discharge catecholamines-containing secretory granules mainly by exocytosis in response to elevation of cytosolic calcium concentration [24,25,26]. The main organelles, which provide regulation of calcium signals triggering exocytosis are rough endoplasmatic reticulum and mitochondria [27,28]. Investigations in recent decades have revealed an essential role of mitochondria in exocytosis and catecholamine secretion [29,30,31]. Two different populations of mitochondria have been found in cytoplasm of adrenomedullary chromaffin cells (perinuclear and subplasmalemmal), and the latter have been shown to provide exocytosis of secretory granules [32]. Our investigation demonstrates reduction of mitochondria content in epinephrine-producing cells and swelling of mitochondria, indicative of their accelerated function, during maturation in the control rats. In most cells mitochondria were found in perinuclear zone. These data suggest that lack of mitochondria, especially in subplasmalemmal region, inhibited epinephrine release to circulation. Numerous reports show that granule-containing cells like adrenomedullary cells, mast cells, basophils and eosinophils release secretory products also by peace-meal degranulation—an alternative pathway, which includes dissolvation of granule content and its molecular release [33,34,35,36,37]. We noted visual enhancement of peace-meal degranulation in postpubertal rats. This suggests that peace-meal degranulation activates to support the secretory process. 

Prenatally and postnatally exposed rats exhibited lower blood levels of epinephrine. Electron microscopy and immunohistochemical reactions revealed changes indicative of lower secretory activity of epinephrine-producing cells. Smaller chromaffin cells, a deficit of mitochondria, fewer secretory granules in the cytoplasm and diminished tyrosine hydroxylase production indicate insufficient epinephrine release. All these changes became more enhanced with age. Lower functional activity of epinephrine-producing cells was also exacerbated by a slowdown in adrenal medulla growth. This resulted in a significant reduction of epinephrine-levels after puberty. A decrease in the growth rate of adrenal medulla were observed only in prenatally and postnatally exposed rats. This began at pubertal age, when we found a slight retardation in the growth rate which became more pronounced in mature animals. These findings indicate that prenatal exposure to low doses of DDT disrupts the adrenal medulla developmental program in the postnatal period. In our previous investigations, we observed an attenuation of adrenocortical zona glomerulosa and zona reticularis postnatal development after prenatal and postnatal exposure to low doses of DDT, associated with impaired transcriptional control of development [38,39]. Taken together, these findings indicate that DDT exerts a dysmorphogenetic effect on the adrenal glands. This is most likely related with interference of DDT with transcriptional regulation of growth, although this hypothesis requires further investigation. 

Postnatally exposed animals demonstrated adequate development of adrenal medulla and insignificant reduction of epinephrine levels in pubertal age. However, these animals showed the greatest decrease in tyrosine hydroxylase production. An evaluation of ultrastructural parameters showed that relatively higher production of epinephrine compared to prenatally and postnatally exposed rats occurred by less reduced total number of mitochondria and as well as their enhanced function, as proved by a swelling of the matrix which was not found in the control and prenatally and postnatally exposed rats. These changes, together with lowered number of secretory granules in cytoplasm, suggest of more efficient epinephrine release to blood circulation. Continued postnatal exposure to the endocrine disrupter led to a sharp decrease in epinephrine production. Immunohistochemical evaluation did not find age-dependent inhibition of tyrosine hydroxylase production, suggesting that more profound decrease in functional activity of adrenomedullary cells was provoked by aggravating insufficiency of secretory machinery. Electron microscopy found similar to control total number of mitochondria and appearance normal and swollen mitochondria indicative of their lower function. Frequently observed peace-meal degranulation and an increased number of secretory granules in the cytoplasm of cells with minimal tyrosine hydroxylase content suggest their accumulation due to impaired discharge from the cells. 

An evaluation of changes induced by DDT showed that the period in which exposure begins plays an important role in the development of catecholamine-producing function. In rats, chromaffinoblasts appear in fetal adrenals on the 18th day of prenatal development, and their formation is complete on the 10th day after birth [40]. Fetal adrenal chromaffin cells produce norepinephrine or dopamine. Epinephrine-producing cells appear on the 3rd day of the postnatal period and increase their number in adrenals until puberty, when they represent 80% of adrenomedullary chromaffin cells [41,42]. These data show that prenatal exposure to DDT might affect migration of chromaffin cell progenitors and development of their secretory apparatus. Slower postnatal development of the adrenal medulla found only in prenatally and postnatally exposed rats confirms this assumption. Delineation of epinephrine- and norepinephrine-producing cells, that is, cells with up-regulated expression of phenylethanolamine N-methyltransferase, an enzyme, that converts norepinephrine to epinephrine, begins after birth and may also be impaired by DDT. In the present investigation we did not observe a decrease in epinephrine-producing cell content in adrenal medulla in all DDT-exposed rats. The main differences between rats with prenatal and postnatal onset of exposure were a level of tyrosine hydroxylase production and changes in mitochondria apparatus. Our results demonstrate that postnatal onset of exposure induces more profound inhibition of tyrosine hydroxylase and impairs its age-related pattern of production. Swelling of mitochondria in pubertal age and the most pronounced reduction in mitochondria content and accumulation of secretory granules in epinephrine-producing cells of the matured rats indicate that postnatal onset of exposure disrupts secretory function to a greater extent. We suppose that the more severe effect of adrenal medulla function in postnatally exposed rats may be associated with the simultaneous action of two stress factors: adaptation to extrauterine life and endocrine disruption. Catecholamines of the central and peripheral nervous system play a significant role in adaptation [43]. In our previous study, we found decreased production of norepinephrine by sympathetic neurons [19]. These data support the idea that impaired catecholamine production by exposure to an endocrine disrupter since birth negatively influences adaptation of neonates and consequently complicates postnatal development of chromaffin-producing function. 

## 4. Materials and Methods

### 4.1. Animals and Experimental Design

Female Wistar rats weighed 180–220 g, (*n* = 15) were obtained from Scientific Center of Biomedical Technologies of Federal Medical and Biological Agency of Russia. The animals were housed at +22–23 °C and given a pelleted standard chow ad libitum. 

The investigation was performed in accordance with the handling standards and rules of laboratory animals as consistent with “International Guidelines for Biomedical Researches with Animals” (1985), laboratory routine standards in the Russian Federation (Order of Ministry of Healthcare of the Russian Federation dated 19.06.2003 No.267) and “Animal Cruelty Protection Act” dated 1.12.1999, regulations of experimental animal operation approved by Order of Ministry of Healthcare of USSR No.577 dated 12.08.1977. Animal procedures were approved by Ethics committee of Research Institute of human morphology (protocol No.28(4), 28 October 2021).

Five female rats received a solution of o,p-DDT 20 µg/L (“Sigma-Aldrich”, St. Louis, MO, USA) ad libitum instead of tap water from the time of mating, during pregnancy and lactation (*n* = 10). Another five female rats received the same solution of DDT only during lactation (*n* = 10). After weaning, the progeny of the rat dams received the same solution of o,p-DDT during postnatal development. The main experimental group included male Wistar rats (*n* = 20) exposed to low doses of o,p-DDT prenatally and postnatally (group DDT E0-P70). A group of male Wistar rats (*n* = 20,) exposed to DDT only during postnatal development (group DDT P0-P70), was included in the experiment to differentiate effects of prenatal exposure. The male offspring (*n* = 22) of 5 intact rat dams was used as a control. Half of the control and exposed rats were sacrificed at 42nd day of postnatal development (P42), which corresponds to pubertal period, when the adrenals actively grow [21]. Other rats were sacrificed on the 70th day of postnatal development (P70), which corresponds to termination of adrenal growth [21]. The rats were sacrificed by zoletil overdosage at 9–10.00 a.m. The average daily DDT consumption by the pregnant dams was 2.70 ± 0.19 µg/kg, the lactating dams—2.47 ± 0.11 µg/kg, by the offspring—3.30 ± 0.14 µg/kg. The received doses correspond to rates of daily dietary exposure of humans to DDT with consideration for differences in DDT metabolism in rats and humans [20,44].

Blood and both adrenal glands were collected. Plasma was separated from EDTA-stabilized blood. One adrenal gland from each rat was processed for light, the other for electron microscopy.

### 4.2. Epinephrine Assay

Epineprine concentration in blood plasma (EDTA) was measured by enzyme-linked immunosorbent assay according to manufacturer’s protocols (Cusabio, Wuhan, China) with “Anthos 2010” microplate reader at 450 nm.

### 4.3. Adrenal Histology

The adrenal glands were fixed in Bouin solution. After standard histological processing the tissue samples were embedded in paraffin. Equatorial sections of the adrenals were stained with hematoxylin and eosin. Histological examination was performed with “Leica DM2500” light microscope (Leica Microsystems Gmbh, Wetzlar, Germany). Histological examination included light microscopy and computer histomorphometry of adrenal medulla. 

### 4.4. Electron Microscopy

The adrenal glands were immediately cut with a thin razor after removal into 1–1.5 mm^3^ pieces under a stereomicroscope and immersed in a fixative containing 1% glutaraldehyde and 0.1 M sodium cacodylate. The tissue samples were postfixed in 1% osmium tetroxide, dehydrated in graded ethanol-acetone series and embedded in a mixture of Epon and Araldite. Semithin sections were studied with light microscopy for selection of areas for ultrathin sectioning. Ultrathin sections were cut with “PowerTomeX” (RMC Boeckeler, Tucson, AZ, USA), counterstained with uranyl acetate and lead citrate and examined with “Libra 120” electron microscope (Carl Zeiss, Ostfildern, Germany). Differentiation of epinephrine- and norepinephrine-producing cells was made on well-known criteria based on distinct fine structure of secretory granules [25]. Only epinephrine-producing cells were enrolled in further ultramorphometrical evaluation.

### 4.5. Computer Histo- and Cytomorphometry

Computer histo- and cytomorphometry of light and electron microscope images were carried out using the “ImageScope” software (Leica Microsystems Gmbh, Wetzlar, Germany). The surface areas of adrenal medulla, cytoplasm of chromaffin cells, nuclei, and mitochondria were measured. The numbers of mitochondria and secretory granules per 1 µm^2^ of cytoplasm, and the total number of mitochondria and secretory granules, were calculated.

### 4.6. Immunohistochemistry

An immunohistochemical evaluation of tyrosine hydroxylase was performed on paraffin-embedded tissues. After antigen retrieval with 10 mM sodium citrate (pH 6.0), endogenous peroxidase and endogenous immunoglobulins were blocked with Hydrogen Peroxide Block and Protein Block (Thermo Fisher Scientific, Waltham, MA, USA). The slides were incubated with primary polyclonal antibodies to rat tyrosine hydroxylase (1: 1000, Abcam, Cambridge, MA, USA) overnight at 8 °C. Slides processed without incubation with primary antibodies were used as a negative control. The reaction was visualized with UltraVision LP Detection System reagent kit (Thermo Fisher Scientific, Waltham, MA, USA) according to manufacturer’s recommendations. The sections were counterstained with Mayer’s hematoxylin.

Percentage of tyrosine hydroxylase-positive and tyrosine hydroxylase-negative chromaffin cells was calculated. Positive cells were subdivided into cells with diffuse and local distribution of tyrosine hydroxylase in the cytoplasm after magnification 1000 microscopy and morphometrical assessment of immunopositive areas. More than 90% of immunopositive cytoplasm was considered a diffuse distribution, and less than 90%—a local distribution. Immunoreactivity intensity distribution index (IRIDI) for tyrosine hydroxylase was calculated as follows: 

IRIDI = the proportion of cells with diffuse distribution of tyrosine hydroxylase in the cytoplasm × 2 + the proportion of cells with local distribution of tyrosine hydroxylase in the cytoplasm × 1 + the proportion of tyrosine hydroxylase-negative cells × 0.

### 4.7. Statistical Analysis

Statistical analyses were carried out using the Statistica 7.0 software package (StatSoft, Tulsa, OK, USA). The central tendency and dispersion of quantitative traits with approximately normal distribution were presented as the mean and standard error of the mean (M ± SEM). Quantitative comparisons of independent groups of the same age were performed using ANOVA. Age-dependent changes in each group were analyzed with a Student’s *t*-test, taking into account the values of Levene’s test for the equality of variances. Quantitative comparisons were performed using Chi-square. Differences were considered statistically significant at *p* < 0.05.

## 5. Conclusions

The results of the present investigation demonstrate that DDT is a potent disrupter of cathecholamines. Low-dose developmental exposure to DDT disrupts adrenal medulla function and impairs epinephrine production. DDT interferes with the synthesis of thyrosine hydroxylase and affects the mitochondrial apparatus of epinephrine-producing cells, which lead to reduced epinephrine secretion during puberty and after maturation. Low-dose exposure to DDT since birth results in more pronounced changes in adrenomedullary cells and a more profound decrease in epinephrine secretion after puberty. Prenatal onset of exposure was found to exert a mild effect on of epinephrine-producing function, but is associated with a lower rate of adrenal medulla growth during maturation, indicative of the dysmorphogenetic action of DDT. The present findings also provide evidence that rat adrenal medulla fails to adapt to DDT exposure and restore proper epinephrine production.

## Data Availability

The data presented in this study are available from the corresponding author upon reasonable request.
